# Disease activity and maternal–fetal outcomes in pregnant women with cushing’s syndrome: a systematic review and meta‑analysis

**DOI:** 10.1007/s11154-026-10016-x

**Published:** 2026-02-04

**Authors:** Diego Barata Bandeira, Gabriela de Abreu Santos, Andrea Glezer, Marcio Carlos Machado, Cesar Luiz Boguszewski, Vania dos Santos Nunes-Nogueira

**Affiliations:** 1 Department of Internal Medicine, Medical School, São Paulo State University (UNESP), Botucatu, São Paulo Brazil; 2 Neuroendocrine Unit, Division of Endocrinology and Metabolism, Hospital das Clínicas, University of Sao Paulo Medical School, São Paulo, São Paulo Brazil; 3 Endocrine Division (SEMPR), Department of Internal Medicine, Federal University of Parana, Curitiba, Brazil

**Keywords:** Cushing’s syndrome, cushing’s disease, ACTH-secreting pituitary adenoma, Ectopic ACTH syndrome, Adrenal cortex hyperfunction, Pregnancy

## Abstract

Pregnancy in women with Cushing’s syndrome (CS) is uncommon due to the detrimental effects of hypercortisolism on fertility and is associated with an increased risk of maternal and fetal complications. This systematic review evaluated the natural course and maternal–fetal outcomes of CS during pregnancy. Searches were conducted in MEDLINE, EMBASE and LILACS for observational studies including at least three pregnant women with CS. Twenty-two studies comprising 381 pregnancies were included. Among 200 women with reported etiologies, 54% had Cushing’s disease, 24% had adrenal adenoma, and 11% had adrenal carcinoma. The overall rate of spontaneous miscarriage was 6% (21 studies, 245 pregnancies; 95% CI, 3–13%), and that of preterm birth was 37% (18 studies, 342 pregnancies; 95% CI, 24–52%). Worsening of preexisting diabetes or development of gestational diabetes was reported in 24% (16 studies, 344 pregnancies; 95% CI, 13–39%), whereas hypertensive disorders of pregnancy (HDP) occurred in 39% (19 studies, 362 pregnancies; 95% CI, 23–58%). Among 134 pregnancies, only one thromboembolic event was reported. The perinatal mortality rate was 7% (18 studies, 342 newborns; 95% CI, 3–14%), low birth weight was 26% (11 studies, 113 newborns; 95% CI, 15–43%), and congenital malformations were 1% (11 studies, 306 newborns; 95% CI, 0–7%). A meta-analysis of four studies revealed that uncontrolled hypercortisolism was associated with higher odds of HDP, diabetes, and preterm birth. These findings suggest that pregnancy in women with uncontrolled CS is associated with an increased risk of adverse maternal and fetal outcomes.

**Supplementary Information:**

The online version contains supplementary material available at 10.1007/s11154-026-10016-x.

## Introduction

Cushing’s syndrome (CS) is a rare and potentially life-threatening disorder caused by chronic cortisol excess and, in extremely rare cases, by increased production of corticotropin-releasing hormone (CRH) [1]. Its prevalence varies across different populations [2], with an estimated annual incidence of 0.7–3.2 cases per million [3]. CS arises from either adrenocorticotropic hormone (ACTH) overproduction or autonomous cortisol secretion by the adrenal glands. When excess ACTH is driven by a pituitary adenoma, the condition is termed Cushing’s disease (CD). Adrenal sources of CS include cortisol-secreting adenomas or carcinomas and, more rarely, adrenal adenomas expressing human chorionic gonadotropin (hCG) receptors that lead to CS exclusively during pregnancy [4]. Rare forms of CS can also result from the ectopic secretion of ACTH by nonpituitary tumors [5].

CS is associated with significant morbidity and mortality, mainly resulting from cardiovascular, metabolic, and skeletal complications, including hypertension, diabetes mellitus, dyslipidemia, coagulopathies, and osteoporosis. Notably, excess mortality may persist even after successful treatment, highlighting the long-term impact of prior hypercortisolism [6].

In addition to its systemic complications, CS is further correlated with reduced fertility, resulting from multiple pathophysiological mechanisms. Chronic excess cortisol suppresses gonadotropin-releasing hormone secretion and decreases the frequency of luteinizing hormone pulses, while the mass effect of pituitary adenomas in CD may further disrupt gonadal function [7, 8]. In addition, elevated androgen levels, particularly in CD, impair ovulatory function and endometrial receptivity, making this form of CS more strongly associated with infertility [9]. Despite these adverse effects on reproductive physiology, pregnancy can still occur in women with CS [10], especially those who have achieved biochemical control of hypercortisolism and/or who have received fertility treatment.

While pregnancy indicates recovery from hypogonadism, concerns persist regarding its bidirectional impact: hypercortisolism may adversely affect pregnancy outcomes, and pregnancy itself may negatively influence the course of CS [11]. Pregnant women with active CS carry significant risks for both the mother and the child, including higher rates of arterial hypertension, preeclampsia, diabetes mellitus, miscarriage, preterm birth, and perinatal mortality, as well as neonatal complications such as low birth weight and small-for-gestational-age infants [8, 12]. Moreover, physiological pituitary hyperplasia during pregnancy, driven by elevated estrogen levels [13], may be particularly concerning in women with macroadenomas. Although this enlargement is typically benign, it can exacerbate mass effects in the presence of a macroadenoma, potentially leading to symptoms such as headache, visual disturbances, or, in rare cases, vision loss [14].

The therapeutic management of CS, particularly in patients with CD, may involve long-term use of medications such as dopamine agonists, somatostatin receptor ligands, or steroidogenesis inhibitors [15]. These agents can improve hypercortisolism and restore fertility, and as a result, pregnancy may occur unexpectedly, often without prior fertility or contraceptive counseling, which might contribute to adverse maternal and fetal outcomes.

Given the clinical relevance of CS and its potential maternal and fetal complications during pregnancy, synthesizing the available evidence is essential to improve the understanding of the disease course and to guide clinical management. Therefore, the objective of this study was to conduct a systematic review of maternal and neonatal outcomes in pregnancies affected by CS, with a particular emphasis on the impact of uncontrolled hypercortisolism on maternal and fetal prognosis.

## Materials and methods

This systematic review was conducted following the Joanna Briggs Institute methodology for systematic reviews of etiology and risk and was reported according to the Preferred Reporting Items for Systematic Reviews and Meta-Analyses [16, 17]. Its protocol was registered in the International Prospective Register of Systematic Reviews under CRD42024597682 and previously published [18], with the complementary description shown below.

### Eligibility criteria

Observational studies (prospective and retrospective cohorts and case series with at least three participants) that met the following participant-exposure of interest-outcome (PEO) structure criteria were included:

#### Participants (P)

Pregnant women of childbearing age diagnosed with CS before, during, or within 12 months after pregnancy.

#### Exposure of interest (E)

CS was diagnosed in accordance with the criteria outlined in current clinical guidelines [1, 19]. Cushing’s disease (CD) was confirmed by at least two abnormal first-line screening tests, elevated plasma ACTH concentrations, and evidence of an ACTH-secreting pituitary adenoma, either through imaging findings or histopathological confirmation following transsphenoidal surgery and subsequent clinical remission. ACTH-independent CS was diagnosed in the presence of suppressed plasma ACTH levels and imaging findings consistent with adrenal pathology, such as adenoma, carcinoma, or bilateral adrenal hyperplasia. Ectopic ACTH secretion was defined by elevated ACTH levels in the absence of a pituitary lesion and identification of an extrapituitary ACTH-producing tumor (e.g., bronchial, or thymic carcinoid, small cell lung carcinoma), confirmed by imaging and/or histopathology.

#### Outcomes (O)

Maternal outcomes were preterm birth, worsening of preexisting diabetes or the development of gestational diabetes, the presence of hypertensive disorders of pregnancy (HDP), spontaneous miscarriage, maternal adverse events related to the use of drugs at conception or during pregnancy, the lactation rate, thromboembolic events and clinical or biochemical remission of CS after pregnancy. In patients with CD, symptomatic tumor growth during pregnancy, manifesting as headache, visual impairment, apoplexy, or surgical intervention, was also evaluated. The fetal and neonatal outcomes were perinatal mortality, low birth weight, low gestational age, and congenital malformation.

We excluded studies with fewer than three participants, owing to scarce data and the potential for imprecision, as well as studies addressing exogenous hypercortisolism (e.g., glucocorticoid therapy), given the distinct pathophysiological mechanisms and prognostic implications compared with those of endogenous disease.

### Identification of studies

#### Electronic databases

Search strategies were created and applied for the following electronic health databases: Embase (by Elsevier), MEDLINE (by PubMed), and Latin American and Caribbean Health Sciences Literature (by the Virtual Health Library). Searches were performed on November 4, 2024, without language or year restrictions. The following index terms and their synonyms were used: “Cushing’s syndrome,” “Pituitary ACTH hypersecretion,” “ACTH-secreting pituitary adenoma,” “Ectopic ACTH syndrome,” “ACTH-secreting adenoma,” “Pregnancy,” and “adrenal cortex hyperfunction” (Supplementary file).

EndNote 20 citation management software was used to download all identified references and remove duplicate entries. The free web application Rayyan was used to perform the initial screening of abstracts and titles [20].

### Study selection, data extraction, and risk of bias evaluation

Two independent reviewers (DBB and GAS) screened the titles and abstracts identified during the literature search, independently read the full-text articles that were potentially eligible, and verified whether these studies met the inclusion criteria via the proposed PEO structure. Both reviewers used a standardized extraction form to assess the data from each study, such as details about exposure (control status before pregnancy, mean age at pregnancy, frequency of neurosurgery before pregnancy, macro- or microadenoma), study design, number of patients, number of pregnancies, and outcome results.

The risk of bias in selected studies was assessed independently by the reviewers via the Checklist for Case Series from the Joanna Briggs Institute Critical Appraisal tools in JBI Systematic Reviews since all the included articles used this study design. This tool checks for clear inclusion criteria, standardization of the criteria, description of CS diagnosis, consecutive and complete inclusion of participants, clarity of clinical and demographic data, complete reporting of outcomes, and appropriate statistical analysis [21].

Any disagreements between the reviewers during the selection or extraction process, as well as in the risk of bias evaluation, were resolved through discussion or by a third reviewer (VSNN).

### Data synthesis and analysis

Similar outcomes among the studies were plotted in the meta-analysis via Stata Statistical Software 18 (Stata Statistical Software: Release 18. College Station, TX, USA).

To calculate the overall proportions of dichotomous data, proportional meta-analyses were performed. We used the updated command metaprop_one and fit the logistic-normal random-effects model to the data [22]. The number of events was used as the numerator, with the total number of pregnancies or newborns serving as the denominator for maternal and fetal outcomes, respectively. To avoid overestimating the rate of CS control and underestimating the proportion of tumor growth during pregnancy in cases of CD, the denominator for these specific outcomes was limited to the number of pregnancies in which the outcomes were explicitly assessed. By employing the logistic-normal random-effects model, the chi-square test (χ²) of the likelihood ratio test comparing the random- and fixed-effects models was used to evaluate the presence of inconsistency. A *p value* < 0.10 was considered indicative of heterogeneity.

For studies assessing the presence of uncontrolled hypercortisolism during pregnancy, we evaluated whether this condition was associated with an increased risk of adverse maternal–fetal outcomes, including abortion, prematurity, perinatal mortality, HDP, and diabetes mellitus. Odds ratios (ORs) were calculated by comparing the odds of these outcomes between pregnant women with uncontrolled hypercortisolism and those with controlled disease. A random-effects meta-analysis was employed [23, 24], with OR as the summary statistic, via the inverse variance pooling method. Inconsistencies in the meta-analyses were ascertained by the I2 statistic, in which an I2 of > 50% indicated a moderate probability of heterogeneity.

In cases of statistical inconsistency among studies and when more than ten studies were included in a meta-analysis, we planned to perform a meta-regression using the metareg command to explore potential sources of variability and better understand the relationships between study characteristics and the evaluated outcomes [25]. Ideally, covariates such as women’s mean age at pregnancy, year of manuscript publication, number of pregnant women taking medications, and, in the cases of CD, number of women who underwent transsphenoidal surgery prior to pregnancy, number of apoplexy events during pregnancy, and number of micro- and macroadenomas should be studied, but most authors did not report these variables, and meta-regression was ultimately performed only using the year of publication as a covariate.

We also incorporate uncertainty in the analyses calculating prediction intervals, which were derived from the random-effects meta-analysis if Chi2 < 0.10 and if more than five studies were included in the analysis [23, 24].

### Quality of evidence

The quality of evidence for the association between uncontrolled hypercortisolism during pregnancy and adverse maternal–fetal outcomes was assessed via the Grading of Recommendations, Assessment, Development, and Evaluation (GRADE) framework for observational studies [26].

## Results

### Study selection

**Fig. 1:**
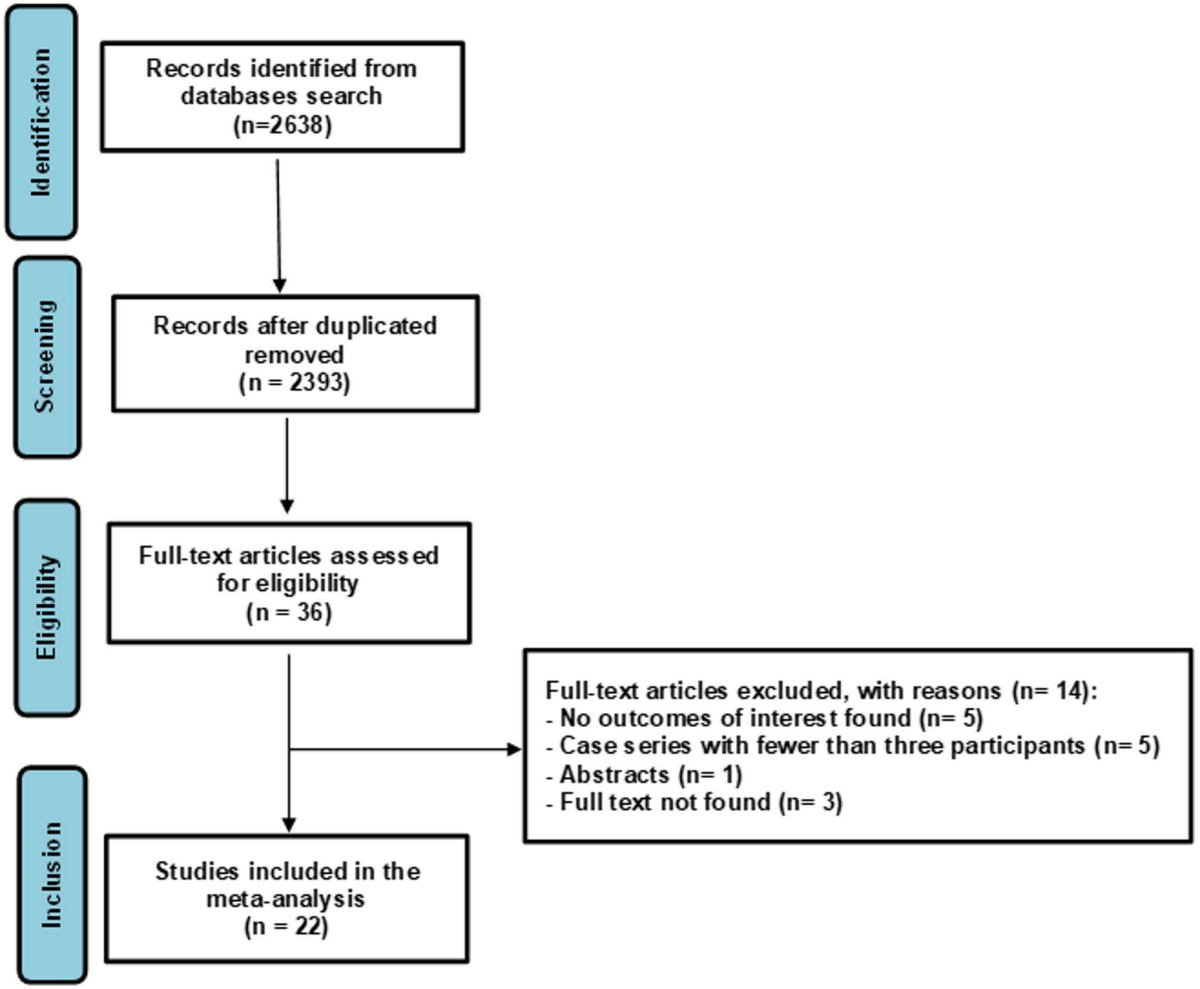
PRISMA flow diagram of the selection of studies

The application of the search strategies resulted in 2638 studies; after removing duplicates, 2393 studies remained. Of these, 36 studies were fully evaluated to assess eligibility; 22 met the inclusion criteria and were included in the review (Fig. 1). The reasons for the exclusion of 14 studies were no outcome of interest (*n* = 5), case series with fewer than three participants (*n* = 5), abstracts (*n* = 1), and full texts not available (*n* = 3). Articles that were not found were retrieved via email, and although a bibliographic switching service was used, 3 references remained inaccessible. The excluded references and their respective justifications are shown in Table 1 of the Supplementary File.

### Characteristics of the included studies

The 22 included articles, published between 1953 and 2024, reported a total of 381 pregnancies [27–48]. Among the 200 women for whom the etiology of CS was reported, 54% had CD, 24% had adrenal adenoma, 11% had adrenal carcinoma, 4% had primary pigmented nodular adrenocortical disease (PPNAD), 2% had ectopic adrenocorticotropic secretion (EAS), and 5% presented with Nelson syndrome. The mean maternal age at pregnancy was 30 years. Information on the timing of CS diagnosis was available in 15 studies: approximately 42% of the diagnoses were made before conception, 24% during pregnancy, and 34% after delivery. The main characteristics of the included participants are summarized in Table 1.

**Table 1:** The main characteristics of participants in the included studies

**Author**	**Year of publication**	**Country**	**Year of the data survey**	**N (w)**	**N (p)**	**Mean age at pregnancy**	**CS Type**	**Controlled Cushing before pregnancy (n)**	**Use medication at conception (n)**	**Use of medication during pregnancy (n)**
Abiven-Lepage	2010	France	1963–2007	12	12	28,8	AC	0	0	0
Andreescu	2017	Belgium		3	3	28	AA	0	0	0
Aron	1990	USA	1978–1986	4	4		AA: 2/PPNAD: 1/EAS: 1			1
Baghlaf	2021	Canada	2004–2014		135					
Cannavo	2011	Italy		3	5		CD		0	0
Carmalt	1977	UK	1964–1973	3	5		CD	5	0	0
Chico	1996	Spain		5	6	32,5	CD	0	0	0
Gaujoux	2020	France	2003–2018	8	8	35,37	PPNAD: 1/CD: 2/AA: 5			
George	2010	Singapore		3	3		AA			
Guilhaume	1992	France		22	22	27,3	AA:8/AC: 8/CD: 4/EAS: 2			
Hochman	2021	France	1989–2020	60	78	29,7	CD	57	0	0
Hunt	1953	USA	1944–1953	7	12		PPNAD: 6/AC: 1	5		
Jornayvaz	2011	France		11	20	30	Nelson syndrome		0	
Juírez-Allen	2013	Argentina		5	5		AA		2	1
Lindsay	2005	USA		4	7	30,75	DC	2	0	1
Odot	2025	France	2008–2023	4	4		DC	0		
Shi	1992	Taiwan		3	3		AA	0		
Stoinis	2024	Australia	2006–2022	5	7	32,8	CD: 4/EAS: 1	1	0	0
Tang	2020	China	2010–2019	19	19	27,68	CD			
Wang	2024	China	2016–2023	5	5	31	AA	0	0	0
Welbourn	1971	Ireland	1953–1968	6	10	27,16	AA			
Zhu	2024	China	2002–2022	8	8	29,87	AA	0		

*N(w)* number of women, *N(p)* number of pregnancies, *CS* Cushing’s syndrome, *AC* adrenal carcinoma, *AA* adrenal adenoma, *PPNAD* primary pigmented nodular adrenocortical disease, *EAS* ectopic adrenocorticotropic hormone secretion, *CD* Cushing’s disease

Only four studies on CD reported whether pituitary surgery was performed and the timing of the procedure (before, during, or after pregnancy). In three of these studies, nearly all surgeries were performed before pregnancy [32, 33, 39]. Only one study (4 pregnancies) reported surgery during pregnancy [41].

In terms of medical therapy, 11 studies reported the use of pharmacological treatment to control hypercortisolism at conception or during pregnancy. Specifically, among 153 pregnancies, two conceptions occurred under ketoconazole treatment, and three women received drug therapy during pregnancy (ketoconazole, metyrapone, and cyproheptadine), with no associated adverse effects reported [29, 40, 41]. In the remaining studies that addressed this outcome, no medical therapy was administered at or during pregnancy.

### Assessment of the risk of bias

Most of the included case series clearly defined standardized inclusion criteria and employed valid methods for identifying CS. They consistently reported clinical information and evaluated outcomes; however, most did not provide relevant demographic details, such as the mean age of the pregnant woman or other risk factors that could influence pregnancy outcomes. Notably, the study with the largest number of participants did not report the underlying cause of CS [30]. Most studies did not report whether patient inclusion was consecutive, and descriptions of the care settings were frequently incomplete. Importantly, for case reports or case series, formal statistical analysis is typically considered “not applicable,” as these studies are descriptive rather than comparative; therefore, the absence of statistical analysis does not constitute a methodological limitation. The risk of bias for each domain across the included studies is presented in the Supplementary File.

### Meta-Analysis

#### Proportion meta-analysis of maternal outcomes

**Fig. 2:**
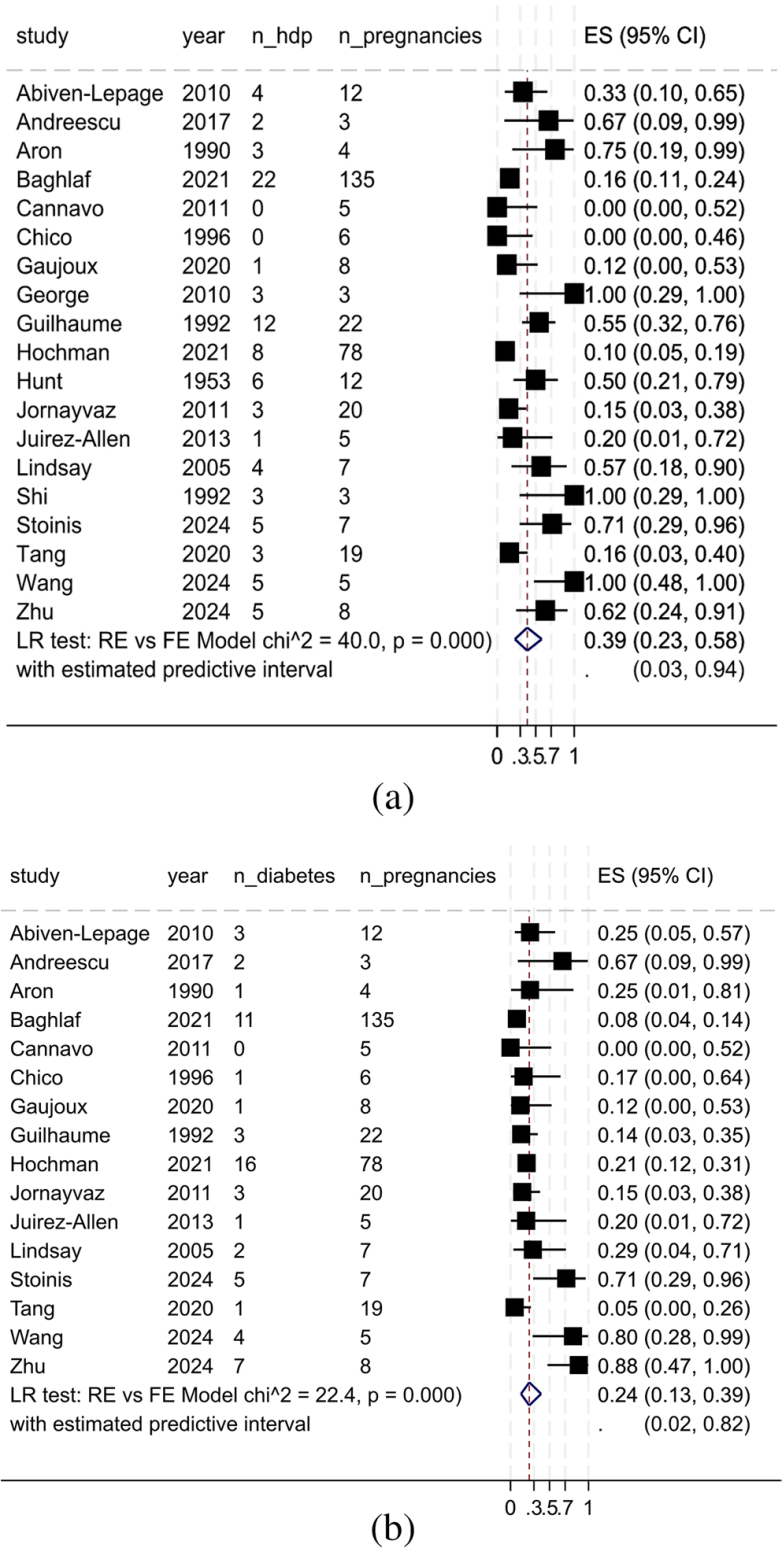
(**a**): Meta-analysis findings: hypertensive disorders during pregnancy. ES, effect size; FE, fixed effects; LR, likelihood ratio; RE, random effects; 95% CI, 95% confidence interval. (**b**): Meta-analysis findings: diabetes. ES, effect size; FE, fixed effects; LR, likelihood ratio; RE, random effects; 95% CI, 95% confidence interval

**Fig. 3:**
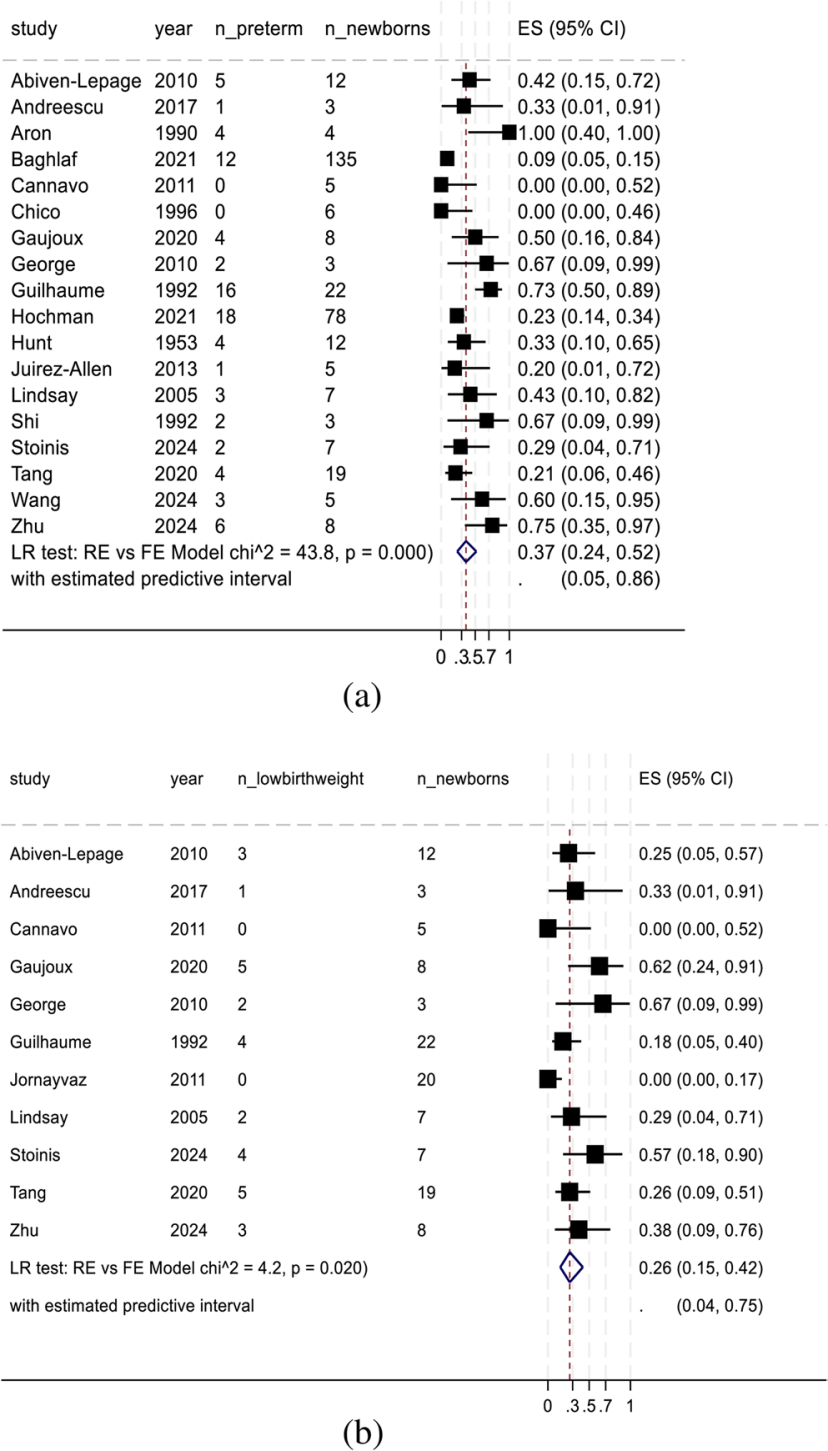
(**a**): Meta-analysis findings: preterm. ES, effect size; FE, fixed effects; LR, likelihood ratio; RE, random effects; 95% CI, 95% confidence interval. (**b**): Meta-analysis findings: low birth weight. ES, effect size; FE, fixed effects; LR, likelihood ratio; RE, random effects; 95% CI, 95% confidence interval

The overall proportion of women with controlled hypercortisolism during pregnancy was 5% (95% CI, 0–46%; 12 studies; 150 women), with substantial heterogeneity across studies. This heterogeneity was driven primarily by outlier reports in which control rates exceeded 70% (Supplementary File). The overall proportion of HDP was 39% (95% CI, 23%–58%; 19 studies; 362 pregnancies; χ² = 40.0; *p* < 0.001; Fig. 2A), whereas the proportion of gestational diabetes or worsening of preexisting diabetes was 24% (95% CI, 13%–39%; 16 studies; 344 pregnancies; χ² = 22.4; *p* < 0.001; Fig. 2B). The overall proportion of preterm newborns was 37% (95% CI, 24%–52%; 18 studies; 342 newborns; χ² = 43.8; *p* < 0.001; Fig. 3A), whereas the proportion of spontaneous abortions was 6% (95% CI, 3%–13%; 21 studies; 245 pregnancies; χ² = 7.8; *p* = 0.003; Supplementary file).

#### Proportion meta-analysis of neonatal outcomes

**Fig. 4:**
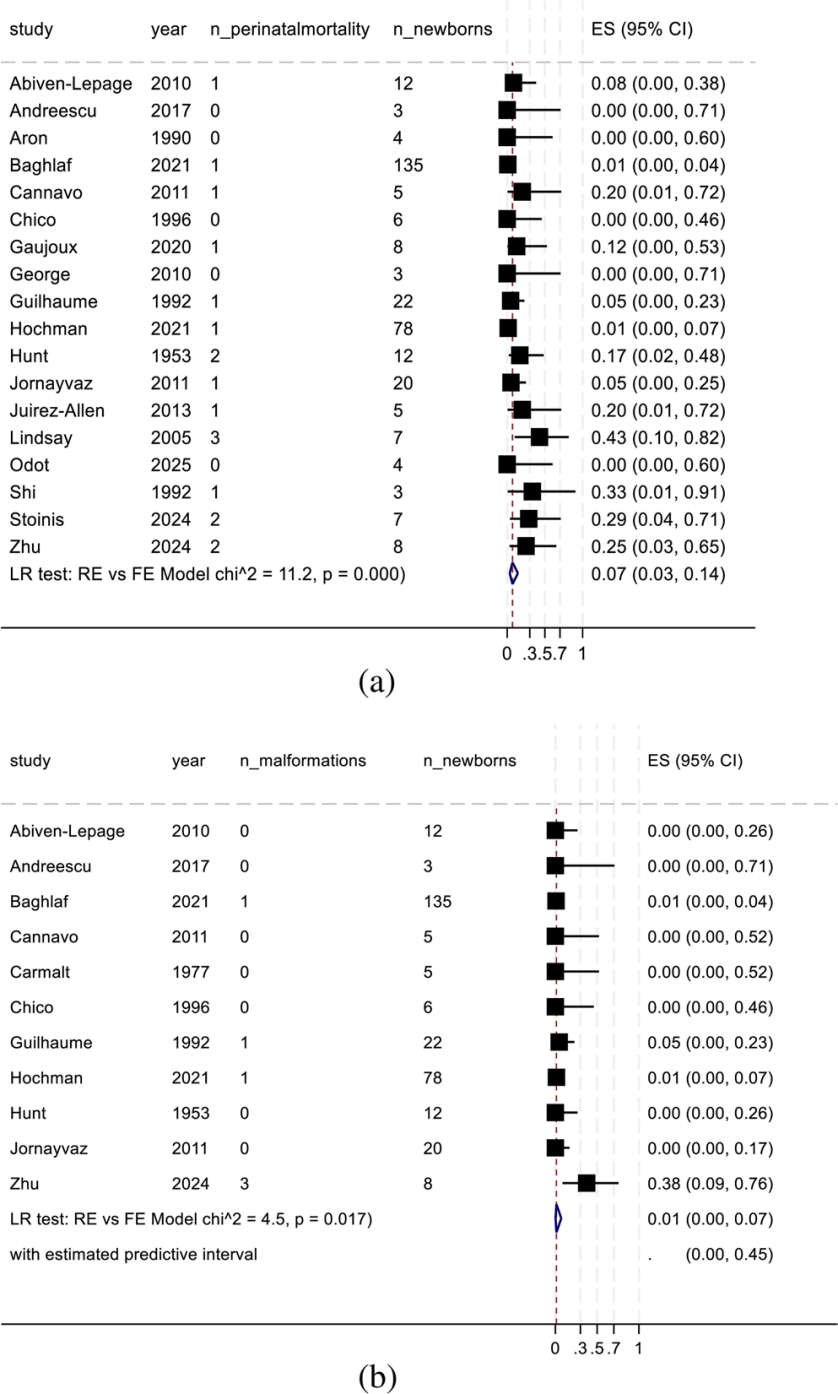
(**a**): Meta-analysis findings: Perinatal mortality. ES, effect size; FE, fixed effects; LR, likelihood ratio; RE, random effects; 95% CI, 95% confidence interval. (**b**): Meta-analysis findings: congenital malformations. ES, effect size; FE, fixed effects; LR, likelihood ratio; RE, random effects; 95% CI, 95% confidence interval

The most frequent perinatal complication was low birth weight, with an overall prevalence of 26% (95% CI, 15–42%; 11 studies; 114 newborns; χ² = 4.2; *p* = 0.02; Fig. 3B). The pooled proportion of perinatal mortality was 7% (95% CI, 3%–14%; 18 studies; 342 newborns; χ² = 11.2; *p* < 0.001; Fig. 4A). Small-for-gestational-age status was observed in 9% of the newborns (95% CI, 3–23%; 8 studies; 196 newborns; χ² = 4.7; *p* = 0.015; Supplementary File). The overall proportion of congenital malformations was 1% (95% CI, 0%–7%; 11 studies; 306 newborns; χ² = 4.5; *p* = 0.017; Fig. 4B).

#### Meta-Analysis of the association between maternal and neonatal outcomes and control of hypercortisolism

Four studies evaluated the impact of hypercortisolism control during pregnancy on maternal and neonatal outcomes [37, 38, 41, 44].

**Fig. 5:**
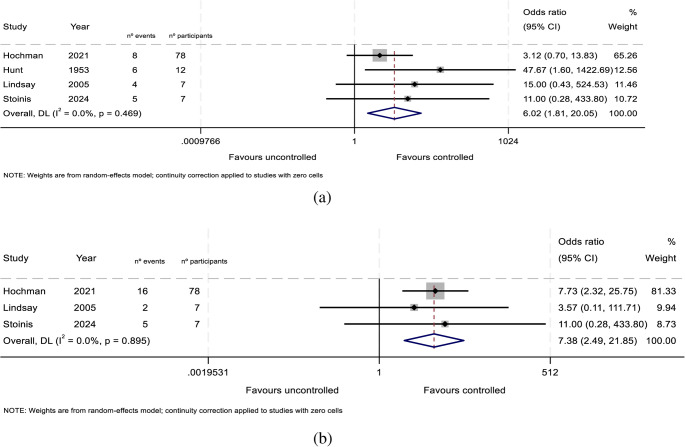
(**a**): Meta-analysis findings: OR hypertensive disorders of pregnancy. ES, effect size; FE, fixed effects; LR, likelihood ratio; RE, random effects; 95% CI, 95% confidence interval. (**b**): Meta-analysis findings: OR diabetes. ES, effect size; FE, fixed effects; LR, likelihood ratio; RE, random effects; 95% CI, 95% confidence interval

For pregnant women with HDP, those with uncontrolled hypercortisolism had a significantly greater risk, with a pooled OR of 6.02 (95% CI, 1.81–20.05; *n* = 104; I² = 0.0%; Fig. 5A; low certainty of evidence, Table 2).

**Table 2:** Summary of findings: association of uncontrolled hypercortisolism during pregnancy with maternal and neonatal outcomes

Certainty assessment							№ of patients		Effect		Certainty	Importance
№ of studies	Study design	Risk of bias	Inconsistency	Indirectness	Imprecision	Other considerations	[Exposure]	[Comparison]	Relative (95% CI)	Absolute (95% CI)		
Prematurity												
4 (104 pregnant women)	nonrandomized studies	serious^a^	not serious	not serious	serious^b^	strong association dose response gradient	27 cases 77 controls		**OR 2.98** (1.14 to 7.78)	-	⨁⨁◯◯ Low^a^,^b^	CRITICAL
							-	17.0%		**209 more per 1.000** (from 19 more to 444 more)		
**Gestational diabetes or worsening of preexisting diabetes**												
3 (92 pregnant women)	nonrandomized studies		not serious	not serious	serious^b^	strong association dose response gradient	23 cases 69 controls		**OR 7.38** (2.49 to 21.85)	-	⨁⨁◯◯ Low^a^,^b^	CRITICAL
							-	10.0%		**351 more per 1.000** (from 117 more to 608 more)		
**Hypertensive disorders during pregnancy**												
4 (104 pregnant women)	nonrandomized studies	serious^a^	not serious	not serious	serious^b^	strong association dose response gradient	23 cases 81 controls		**OR 6.02** (1.81 to 20.05)	-	⨁⨁◯◯ Low^a^,^b^	CRITICAL
							-	6.0%		**218 more per 1.000** (from 44 more to 501 more)		
**Abortion**												
2 (90 pregnant women)	nonrandomized studies	serious^a^	not serious	not serious	very serious^b^,^c^	none	3 cases 87 controls		**OR 2.23** (0.19 to 26.62)	-	⨁◯◯◯ Very low^a^,^b^	CRITICAL
							-	1.6%		**19 more per 1.000** (from 13 fewer to 286 more)		
**Perimortality**												
3 (97 pregnant women)	nonrandomized studies	serious^a^	not serious	not serious	very serious^b^,^c^	none	6 cases 0 controls		**OR 6.65** (0.98 to 44.94)	-	⨁◯◯◯ Very low^a^,^b^,^c^	CRITICAL
							-	0.0%		**0 fewer per 1.000** (from 0 fewer to 0 fewer)		

*CI* confidence interval, *OR* odds ratio

Explanations

a. Nonconsecutive patient admission

b. Small sample size and number of events

c. Confidence interval crosses the threshold of interest

For diabetes mellitus, the meta-analysis demonstrated an even stronger association, with an OR of 7.38 (95% CI, 2.49–21.85; *n* = 92; I² = 0.0%; Fig. 5B; low certainty of evidence; Table 2), indicating a markedly increased risk in the presence of poor hypercortisolism control.

For preterm birth, uncontrolled CS was also associated with increased odds, with an OR of 2.98 (95% CI, 1.14–7.78; *n* = 104; I² = 0.0%; low certainty of evidence, Supplementary file).

In contrast, the association between uncontrolled CS and perinatal mortality had an OR of 6.65 (95% CI, 0.98–44.94; *n* = 97; I² = 0.0%; very low certainty of evidence, Supplementary file), suggesting a possible increased risk, although statistical significance was not reached. Similarly, the pooled analysis for miscarriage yielded an OR of 2.23 (95% CI, 0.19–26.62; *n* = 90; I² = 19.9%; very low certainty of evidence, Supplementary file), with no statistically significant association.

#### Outcomes not reported in quantitative synthesis

None of the included studies reported outcomes related to symptomatic tumor growth during pregnancy in women with CD, such as headache, visual impairment, or pituitary apoplexy. Data regarding breastfeeding practices and outcomes were not available. Among the 134 pregnancies in which thromboembolic events were specifically assessed, only one case was documented. Maternal death was reported in one case of CS, as described in the controlled study by Baghlaf et al. (2022) [30], corresponding to a rate of 700 per 100,000 births. In contrast, the control group reported 637 maternal deaths, with a markedly lower rate of 7 per 100,000 births.

## Discussion

This systematic review and meta-analysis aimed to clarify the impact of CS on maternal and fetal outcomes during pregnancy. We observed that only a minority of women were receiving off-label CS-directed pharmacological treatment at conception or continued/resumed therapy during gestation, and no serious adverse drug reactions were reported. Among neonates, the most frequent adverse outcome was low birth weight, followed by small-for-gestational-age and perinatal mortality, whereas congenital malformations were uncommon. Maternal outcomes were markedly different from those in women without CS, with higher rates of diabetes exacerbation, HDP, and preterm birth. A single case of thromboembolic event and another of maternal death were reported.

Of the 22 included studies, 15 described the timing of the CS diagnosis. The frequency of diagnosis was approximately 42% before conception, 24% during the gestational period, and 34% after gestation. The predominance of diagnoses occurring during or after gestation may help explain the low rate of disease control observed in this cohort, as well as the limited use of CS-directed pharmacological treatment at conception or during pregnancy.

When our findings were compared with those previously reported in the literature, Caimari et al. (2017) conducted a systematic review that included 220 patients with active CS across 263 pregnancies between 1952 and 2015 [49]. In that cohort, 44.1% of cases were attributed to adrenal adenomas. In contrast, our review identified CD as the underlying etiology in 54% of hypercortisolism cases during pregnancy. Notably, our analysis incorporated more recent publications, including larger case series published after 2015, and applied stricter inclusion criteria by considering only studies that reported on at least three pregnant women with CS; these criteria were not used in the previous review. Additionally, we performed a proportional meta-analysis of key maternal and neonatal outcomes, as well as a meta-analysis evaluating the associations between hypercortisolism and adverse maternal and fetal outcomes; these analyses were not previously conducted.

To further expand our analysis, we also evaluated all available case reports and case series, including at least two pregnant women diagnosed with CS, totaling 249 pregnancies not included in our primary dataset. Among these, adrenal adenoma was the most frequently reported etiology (125 cases), followed by CD (70 cases), adrenocortical carcinoma (24 cases), EAS (12 cases), and PPNAD (2 cases). An additional 16 cases were attributed to less common etiologies, including Carney complex, nodular adrenal hyperplasia, and aberrant hormone receptor activation, or were insufficiently specified. Even if these additional cases were included in our analysis, the frequency of CD would remain slightly higher than that of CS due to adrenal adenoma (39.6% versus 38.5%, respectively). Notably, the study with the largest population (135 pregnant women) did not report the underlying cause of CS, which limits comparisons across etiologies [30].

With respect to HDP, long-term hypercortisolism in patients with CS is associated with various complications, such as hypertension and hyperglycemia. The prevalence of hypertension in individuals with endogenous CS is 80% in adults and can reach 95% with EAS [50], and its pathogenesis involves different mechanisms related to plasma volume regulation, peripheral vascular resistance, and cardiac output, in addition to mineralocorticoid activity and activation of the renin‒angiotensin system by excess cortisol [51–53]. During pregnancy, hypertension is the main risk factor for the development of preeclampsia/eclampsia and is independently associated with negative maternal and fetal outcomes [54]. In our review, a frequency of 39% for HDP (hypertension/preeclampsia/eclampsia) was described. In the general population, hypertension occurs in 8–10% of pregnancies, while the prevalence of preeclampsia varies between 2% and 8% [55–57].

Hypercortisolism is also associated with a chronic increase in blood glucose due to stimulation of gluconeogenesis and reduced insulin sensitivity [58]. These factors, combined with gestational changes, such as increased secretion of anti-insulin hormones, chronic insulin resistance, and β-cell dysfunction, explain 23% of the abnormalities in glucose homeostasis among pregnant women with CS. This value is higher than that described for the general population, where the frequency of gestational diabetes or worsening of preexisting diabetes ranges from 10.9 to 16.9% [59–62].

In our review, the frequency of miscarriage was 6%, which is surprisingly lower than the frequency described in the general population, which ranges from 10 to 15.3% in different series [63, 64]. Andersen et al. (2000) reported that maternal age is a crucial determinant factor, with older women having a higher risk of miscarriage, especially due to fetal chromosomal abnormalities [65]. Among the studies included in our meta-analysis that presented these data, the low average age (30 years) may be one of the justifications for the lower incidence of miscarriage in the evaluated population. However, alternative explanations should also be considered, including potential underreporting of very early pregnancies, the inclusion of only clinically recognized pregnancies, and selective publication of successful or ongoing pregnancies. These factors may have contributed to an underestimation of the true miscarriage rate in the analyzed studies.

Perinatal mortality is an important indicator of the quality of obstetric and neonatal care, reflecting social and structural inequalities between different regions of the world. With respect to the global perinatal mortality rate, a frequency of 36 deaths per 1000 live births has been described [66, 67]. In countries with a higher human development index, this rate may be less than 1%, whereas in low-income countries, especially in sub-Saharan Africa, rates exceed 4% to 6% [68]. In the present review, the reported perinatal mortality rate was 7%, despite most included studies being conducted in high-income countries. This finding raises the possibility that hypercortisolism may contribute to increased perinatal mortality, even in settings with otherwise favorable maternal and neonatal care standards.

The overall proportion of low birth weight pregnancies affected by CS was 27%, which is closely related to the high prematurity rate of 36% reported in this review. The “Baby-Cush” study [37], which compared pregnancy outcomes according to maternal cortisol status, reported that the absence of eucortisolism during pregnancy—including hypocortisolism—was associated with an increased risk of prematurity and maternal‒fetal comorbidities compared with the general population. In contrast, prematurity rates in the general population range from 9.9% to 10.4% [69, 70], and the rate of low birth weight is estimated at 14.7%, highlighting the substantial impact of maternal CS on neonatal outcomes [71].

Proportions of 1% for congenital malformation and 9% for small for gestational age were reported in this study, which are lower than those described in the general population, reaching 11% and 27%, respectively [72, 73].

To our knowledge, this is the first meta-analytic assessment specifically evaluating the impact of hypercortisolism control on maternal and neonatal outcomes in pregnancies complicated by CS. Our analysis revealed that uncontrolled hypercortisolism might be associated with an increased risk of HDP and diabetes during pregnancy, with pooled odds ratios of 6.02 and 7.38, respectively. These findings underscore the critical importance of disease control in mitigating maternal morbidity. The observed association with preterm birth further highlights the potential adverse effects of persistent hypercortisolism on fetal outcomes, emphasizing that inadequate disease management during gestation may compromise perinatal health.

While trends toward increased perinatal mortality and miscarriage were noted in women with uncontrolled CS, these associations did not reach statistical significance, likely reflecting the limited number of studies and small sample sizes. These results reinforce the clinical imperative to monitor and manage cortisol levels throughout gestation to optimize both maternal and neonatal outcomes.

When the associations between pregnancy and CS and other pituitary disorders are compared, important differences emerge. Although a direct comparison is not possible owing to variations in disease pathophysiology and study design, outcomes reported for pregnancies in women with acromegaly and prolactinoma appear broadly similar to those reported in the general population [74, 75]. Meta-analyses of these conditions revealed low frequencies of adverse events: in acromegaly, gestational diabetes occurred in 9%, hypertensive disorders in 6%, and prematurity in 9%, whereas prolactinoma studies reported miscarriage at 10%, prematurity at 3%, gestational diabetes at 4%, and congenital malformations at 2%. These rates contrast sharply with those reported in CS, where our review revealed gestational diabetes in 24% and hypertensive disorders in 39%, alongside higher risks of prematurity and low birth weight. A key distinction is that most women with acromegaly or prolactinoma conceived after achieving control of tumor activity and hormonal hypersecretion, often through medical or surgical treatment, whereas pregnancies complicated by CS frequently occurred under conditions of uncontrolled hypercortisolism, which likely increases metabolic and vascular risks.

The main limitations of our systematic review stem from the study designs and small sample sizes of the included articles, reflecting the rarity of CS during pregnancy. Within this context, our meta-analysis demonstrated markedly higher rates of metabolic and hypertensive complications during pregnancy. In contrast, Baghlaf et al. (2021) [30], the only controlled study using a large population-based cohort, reported no significant increase in gestational diabetes risk among CS patients compared with controls (8.1% vs. 5.8%, p = NS) and reported a substantially lower prevalence of preeclampsia (8.1%) than previously documented. They also observed four cases of small-for-gestational-age infants (3%) compared with 2.2% in controls, with no differences between groups regarding this and other fetal outcomes, such as intrauterine fetal death and congenital anomalies. These discrepancies likely reflect differences in study design and case ascertainment: earlier reports were based on uncontrolled series, often including patients with active disease, whereas Baghlaf’s cohort may have included women in remission or under treatment, mitigating metabolic and vascular risks. Additionally, the inability to evaluate outcomes according to disease etiology and specific medical treatments represents an additional important limitation.

Although it is plausible that hypercortisolism is associated with adverse pregnancy outcomes, it is important to emphasize that many of the included studies were conducted before the widespread availability of high-resolution pituitary imaging, standardized referral pathways, advances in pituitary surgery, and modern obstetric and perinatal care. Consequently, complication rates reported in these earlier settings may be higher than those observed in contemporary clinical practice, potentially inflating the pooled estimates presented here. Moreover, the large effect sizes observed may reflect residual confounding, particularly differences in access to specialized care among women with controlled disease, rather than a purely causal relationship. Finally, the small number of included studies and limited sample sizes may have further contributed to effect size inflation, underscoring the need for cautious interpretation of these findings.

In conclusion, pregnancy in women with CS appears to be associated with an increased risk of adverse maternal and fetal outcomes, including higher rates of prematurity, hypertensive disorders of pregnancy, worsening of preexisting diabetes or the development of gestational diabetes, and low birth weight. These risks seem to be more frequent in cases of uncontrolled hypercortisolism, in which active disease may exacerbate metabolic and vascular complications. However, the certainty of the available evidence is low, primarily due to small sample sizes and risk of bias. Despite these limitations, early diagnosis of CS and effective management aimed at achieving disease control remain important strategies for potentially improving maternal and neonatal outcomes.

## Supplementary Information

Below is the link to the electronic supplementary material.

Supplementary Material 1

## Data Availability

No datasets were generated or analysed during the current study.
