# Supplementary material for: Disease activity and maternal–fetal outcomes in pregnant women with cushing’s syndrome: a systematic review and meta‑analysis
Source: Rev Endocr Metab Disord. 2026 Feb 4;27(2):255–68. doi: 10.1007/s11154-026-10016-x (PMC13167853; doi:10.1007/s11154-026-10016-x)
Supplement: Supplementary file 1 — Supplementary Material 1 [file 11154_2026_10016_MOESM1_ESM.docx]

**Disease activity and maternal–fetal outcomes in pregnant women with Cushing’s syndrome: a systematic review and meta‑analysis**

**Supplementary file**

Diego Barata Bandeira^1^; Gabriela de Abreu Santos ^1^; Andrea Glezer^2^; Cesar Luiz Boguszewski^3^; Marcio Carlos Machado^2;^ Vania dos Santos Nunes-Nogueira^1^

^1^Department of Internal Medicine, São Paulo State University (UNESP), Medical School, Botucatu, São Paulo, Brazil.

^2^Neuroendocrine Unit, Division of Endocrinology and Metabolism, Hospital das Clínicas, University of Sao Paulo Medical School, São Paulo, Brazil.

^3^Endocrine Division (SEMPR), Department of Internal Medicine, Federal University of Parana, Curitiba, Brazil.

**Corresponding Author information:**

Nunes-Nogueira VS, PhD, MD

Department of Internal Medicine, São Paulo State University (UNESP), Medical School, Brazil, Sao Paulo, Botucatu.

Email: vania.nunes-nogueira@unesp.br

**Table I.** Excluded studies and reasons…………………………………....……………………….….3

**Table II.** Risk of bias evaluation of included studies.………………………………………….….…4

**Figure I.** Meta-analysis findings: Frequency of participants with controlled disease…….………….6

**Figure II.** Meta-analysis findings: Frequency of miscarriage……………………….……………….7

**Figure III**. Meta-analysis findings: Frequency of small for gestational age….……...……………....8

**Figure IV**. Meta-analysis findings: Odds ratio of preterm…..………………………………….……9

**Figure V**. Meta-analysis findings: Odds ratio of perinatal mortality…………………………….…10

**Figure IV**. Meta-analysis findings: Odds ratio of miscarriage………………………………………11

**Search Strategies**…………………………………………………………………………………...12

**Table I.** Excluded studies and reasons

| **Study** | **Year of publication** |  | **Country** | **Reasons** |
| --- | --- | --- | --- | --- |
| Billaud | 1992 |  | France | Not found. |
| Conger | 2017 |  | Netherlands | Not found. |
| Ferrau | 2012 |  | Italy | Case series with less than 3 participants. |
| Ilie | 2019 |  | Romania | Case series with less than 3 participants. |
| Jia | 2023 |  | China | No outcomes of interest. |
| Jorgensen | 1973 |  | Iran | Case series with less than 3 participants. |
| Kasperlik-Zaluska | 2000 |  | Poland | Case series with less than 3 participants. |
| Khalimova | 2020 |  | Uzbekistan | No outcomes of interest. |
| Lousada | 2022 |  | Brazil | No outcomes of interest. |
| Manusharova | 1990 |  | Russia | Not found. |
| Palejwala | 2018 |  | USA | No outcomes of interest. |
| Rees | 2016 |  | UK | No outcomes of interest. |
| Roper | 2023 |  | Canada | Abstract |
| Shah | 2024 |  | India | Case series with less than 3 participants. |

**Table II.** Risk of bias evaluation of included studies.

| **Author** | **Year** | **1. Were there clear criteria for inclusion in the case series?** | **2. Was the condition measured in a standard, reliable way for all participants included in the case series?** | **3. Were valid methods used for identification of the condition for all participants included in the case series?** | **4. Did the case series have consecutive inclusion of participants?** | **5. Did the case series have complete inclusion of participants?** | **6. Was there clear reporting of the demographics of the participants in the study?** | **7. Was there clear reporting of clinical information of the participants?** | **8. Were the outcomes or follow up results of cases clearly reported?** | **9. Was there clear reporting of the presenting site(s)/clinic(s) demographic information?** | **10. Was appropriate statistical analysis used?** |
| --- | --- | --- | --- | --- | --- | --- | --- | --- | --- | --- | --- |
| Abiven-Lepage | 2010 | Y | Y | Y | Y | Y | N | Y | Y | Y | Y |
| Andreescu | 2017 | Y | Y | Y | N | Y | N | Y | Y | N | NA |
| Aron | 1990 | Y | N | Y | N | Y | N | N | N | N | NA |
| Cannavo | 2011 | Y | U | Y | N | N | N | N | Y | N | NA |
| Carmalt | 1977 | Y | Y | Y | N | N | N | N | N | N | NA |
| Chico | 1996 | Y | Y | Y | N | U | N | Y | Y | N | NA |
| Gaujoux | 2020 | Y | Y | Y | N | N | Y | Y | N | N | NA |
| George | 2010 | Y | Y | Y | N | N | N | Y | Y | N | NA |
| Guilhaume | 1992 | U | N | N | N | N | N | N | Y | N | NA |
| Hochman | 2021 | Y | Y | Y | Y | Y | N | Y | Y | Y | Y |
| Hunt | 1953 | Y | Y | Y | N | N | N | Y | Y | N | NA |
| Jornayvaz | 2011 | Y | Y | Y | Y | Y | N | Y | Y | N | NA |
| Juírez-Allen | 2013 | Y | U | Y | Y | Y | N | Y | Y | N | NA |
| Lindsay | 2005 | Y | Y | Y | N | N | N | Y | Y | N | NA |
| Odot | 2025 | Y | Y | Y | N | N | N | N | N | N | Y |
| Shi | 1992 | U | U | U | N | N | N | Y | Y | N | NA |
| Stoinis | 2024 | Y | Y | Y | Y | Y | N | Y | Y | N | NA |
| Tang | 2020 | Y | Y | Y | N | N | N | Y | Y | N | Y |
| Wang | 2024 | Y | Y | Y | N | Y | N | Y | Y | N | NA |
| Welbourn | 1971 | Y | Y | Y | Y | Y | N | Y | Y | N | NA |
| Zhu | 2024 | Y | Y | Y | Y | Y | Y | Y | Y | N | NA |

| **Author** | **Year** | **1. Were the groups comparable other than the presence of disease in cases or the absence of disease in controls?** | **2. Were cases and controls matched appropriately?** | **3. Were the same criteria used for identification of cases and controls?** | **4. Was exposure measured in a standard, valid and reliable way?** | **5. Was exposure measured in the same way for cases and controls?** | **6. Were confounding factors identified?** | **7. Were strategies to deal with confounding factors stated?** | **8.Were outcomes assessed in a standard, valid and reliable way for cases and controls?** | **9. Was the exposure period of interest long enough to be meaningful?** | **10. Was appropriate statistical analysis used?** |
| --- | --- | --- | --- | --- | --- | --- | --- | --- | --- | --- | --- |
| Baghlaf | 2021 | Y | N | Y | Y | Y | Y | Y | Y | Y | Y |

**Figure I.** Meta-analysis findings: Frequency of participants with controlled disease.

**Figure II.** Meta-analysis findings: Frequency of miscarriage

**Figure III**. Meta-analysis findings: Frequency of small for gestational age

**Figure IV**. Meta-analysis findings: Odds ratio of preterm


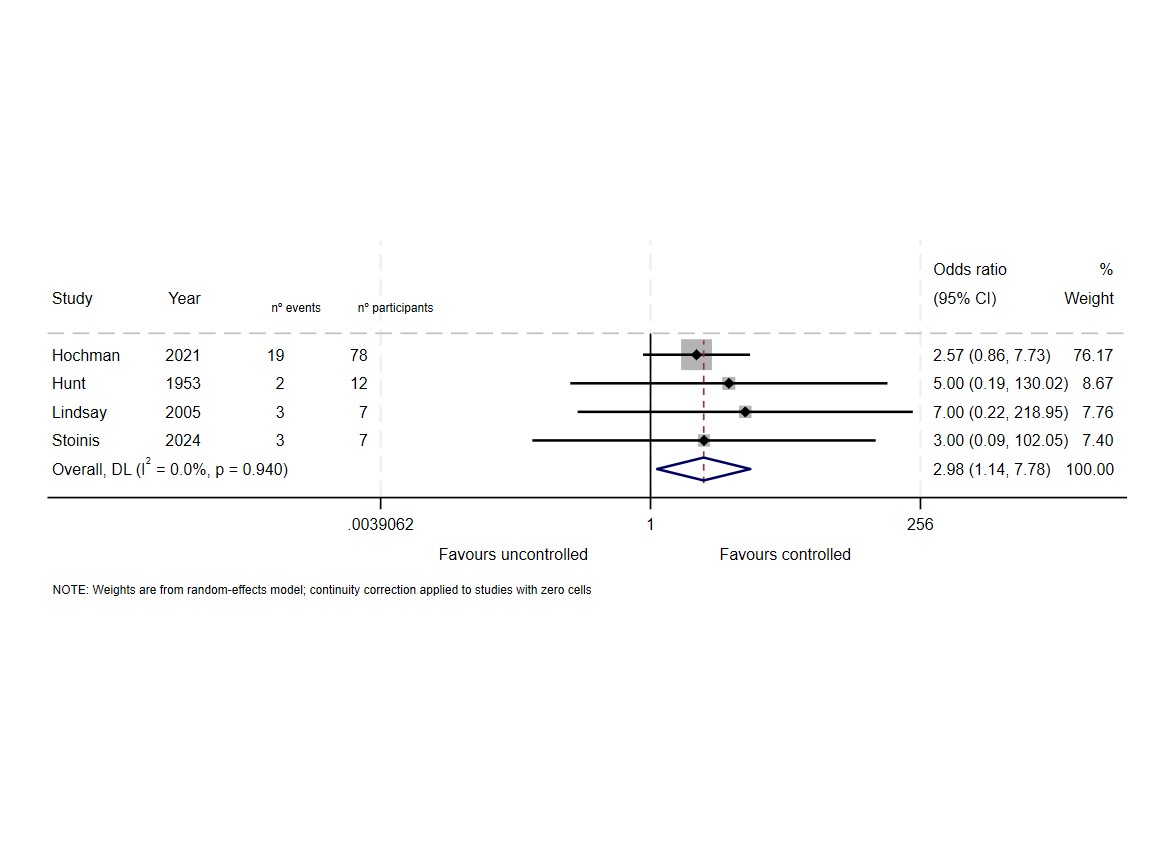


**Figure V**. Meta-analysis findings: Odds ratio of perinatal mortality

**Figure IV**. Meta-analysis findings: Odds ratio of miscarriage

**SEARCH STRATEGIES**

04/November/2024

**1) PubMed**

#1 “Pituitary ACTH Hypersecretion” [Mesh] OR (ACTH Hypersecretion, Pituitary) OR (Hypersecretion, Pituitary ACTH) OR (Cushing Disease, Pituitary) OR (Cushing Diseases, Pituitary) OR (Pituitary Cushing Disease) OR (Pituitary Cushing Diseases) OR (Pituitary-Dependant Hypercortisolism Disorder) OR (Hypercortisolism Disorder, Pituitary-Dependant) OR (Hypercortisolism Disorders, Pituitary-Dependant) OR (Pituitary Dependant Hypercortisolism Disorder) OR (Pituitary-Dependant Hypercortisolism Disorders) OR (Pituitary-Dependant Cushing Syndrome) OR (Cushing Syndrome, Pituitary-Dependant) OR (Pituitary Dependant Cushing Syndrome) OR (Pituitary-Dependant Hypercortisolism) OR (Hypercortisolism, Pituitary-Dependant) OR (Hypercortisolisms, Pituitary-Dependant) OR (Pituitary Dependant Hypercortisolism) OR (Pituitary-Dependant Hypercortisolisms) OR (Cushing Disease) OR (Disease, Cushing) OR (Pituitary Cushing Syndrome) OR (Cushing Syndrome, Pituitary) OR (Inappropriate ACTH Secretion Syndrome) OR (Inappropriate Adrenocorticotropic Hormone Secretion) OR (Adrenocorticotropic Hormone, Inappropriate Secretion)

#2 “Cushing Syndrome”[Mesh] OR (Syndrome, Cushing) OR (Cushing’s Syndrome) OR (Syndrome, Cushing’s) OR (Hypercortisolism) OR ACTH Secreting Pituitary Adenoma OR “ACTH-Secreting Pituitary Adenoma”[Mesh] OR (ACTH-Secreting Pituitary Adenomas) OR (Pituitary Adenomas, ACTH-Secreting) OR (Corticotroph Adenoma) OR (Adenoma, Corticotroph) OR (Adenomas, Corticotroph) OR (Corticotroph Adenomas) OR (Pituitary Corticotropin-Secreting Adenoma) OR (Corticotropin-Secreting Adenoma, Pituitary) OR (Corticotropin-Secreting Adenomas, Pituitary) OR (Pituitary Corticotropin Secreting Adenoma) OR (Pituitary Corticotropin-Secreting Adenomas) OR (ACTH-Producing Pituitary Adenoma) OR (ACTH Producing Pituitary Adenoma) OR (ACTH-Producing Pituitary Adenomas) OR (Pituitary Adenoma, ACTH-Producing) OR (Pituitary Adenomas, ACTH-Producing) OR (Pituitary Adenoma, ACTH-Secreting) OR (Pituitary Adenoma, ACTH Secreting)

#3 "ACTH Syndrome, Ectopic"[Mesh] OR (Ectopic ACTH Syndrome) OR (ACTH Syndromes, Ectopic) OR (Ectopic ACTH Syndromes) OR (Syndrome, Ectopic ACTH) OR (Syndromes, Ectopic ACTH)

#4 “Pregnancy” [Mesh] OR (Pregnancies) OR (Gestation) OR (Pregnant women) OR (Pregnant) OR (Lactating women) OR (Maternal iodine intake) OR (Postpartum) OR (Pregnant patient)

#1 OR # 2 OR #3 AND #2 = 1421

**2) EMBASE**

**#**1 'Cushing syndrome'/exp OR 'adrenal cortex hyperplasia' OR 'adrenal cortical hyperplasia' OR 'adrenocortical hyperplasia' OR 'adrenocorticohyperplasia' OR 'arenocortical hyperplasia' OR 'Cushing`s syndrome' OR 'Cushings syndrome' OR 'endogenous hypercortisolism' OR 'Itsenko-Cushing syndrome' OR 'Itsenko-Cushing`s syndrome' OR 'pituitary basophilism' OR 'Cushing syndrome'

#2' Cushing disease'/exp OR 'adrenocortical hyperplasia, acth induced' OR 'corticotroph pituitary adenoma' OR 'corticotropin induced adrenocortical hyperplasia' OR 'cushing syndrome, acth induced' OR 'Cushing`s disease' OR 'Cushings disease' OR 'Itsenko Cushing disease' OR 'Itsenko Cushing`s disease' OR 'pituitary ACTH hypersecretion' OR 'pituitary corticotroph microadenoma' OR 'pituitary Cushing syndrome' OR 'pituitary Cushing`s syndrome' OR 'pituitary-dependent Cushing disease' OR 'pituitary-dependent Cushing syndrome' OR 'pituitary-dependent Cushing`s disease' OR 'pituitary-dependent Cushing`s syndrome' OR 'pituitary-dependent hypercortisolism' OR 'Cushing disease'

#3 'ACTH secreting adenoma'/exp OR 'ACTH producing adenoma' OR 'ACTH producing adenomas' OR 'ACTH producing pituitary adenoma' OR 'ACTH producing pituitary adenomas' OR 'ACTH producing pituitary tumor' OR 'ACTH producing pituitary tumors' OR 'ACTH producing pituitary tumour' OR 'ACTH producing pituitary tumours' OR 'ACTH producing tumor' OR 'ACTH producing tumors' OR 'ACTH producing tumour' OR 'ACTH producing tumours' OR 'ACTH secreting adenomas' OR 'ACTH secreting pituitary adenoma' OR 'ACTH secreting pituitary adenomas' OR 'ACTH secreting pituitary tumor' OR 'ACTH secreting pituitary tumors' OR 'ACTH secreting pituitary tumour' OR 'ACTH secreting pituitary tumours' OR 'ACTH secreting tumor' OR 'ACTH secreting tumors' OR 'ACTH secreting tumour' OR 'ACTH secreting tumours' OR 'ACTH-secreting pituitary adenoma' OR 'adrenocorticotropic hormone secreting adenoma' OR 'corticotroph adenoma' OR 'corticotroph adenomas' OR 'corticotropinoma' OR 'corticotropinomas' OR 'ACTH secreting adenoma'

#4 'adrenal cortex hyperfunction'/exp OR 'adrenal gland hyperfunction' OR 'adrenocortical hyperfunction' OR 'hyperadrenocorticalism' OR 'hyperadrenocorticism' OR 'hypercorticism' OR 'adrenal cortex hyperfunction'

#5 'ectopic corticotropin production'/exp OR 'acth syndrome, ectopic' OR 'corticotropin syndrome' OR 'ectopic ACTH secreting tumor' OR 'ectopic ACTH secretion' OR 'ectopic ACTH secretion syndrome' OR 'ectopic ACTH syndrome' OR 'ectopic corticotropin secretion' OR 'ectopic corticotropin syndrome' OR 'ectopic production of corticotropin' OR 'ectopic secretion of ACTH' OR 'ectopic secretion of corticotropin' OR 'occult ectopic ACTH secretion' OR 'ectopic corticotropin production'

#6 ‘pregnancy’/exp OR ‘child bearing’ OR ‘childbearing’ OR ‘gestation’ OR ‘gravidity’ OR ‘intrauterine pregnancy’ OR ‘labor presentation’ OR ‘labour presentation’ OR ‘pregnancy maintenance’ OR ‘pregnancy trimesters’

(#1 OR #2 OR #3 OR #4 OR #5) AND #6 = 1202

**3) Lilacs (BVS)**

#1 MH: “Hipersecreção Hipofisária de ACTH” OR (Hipersecreção de ACTH Hipofisária) OR (Hipersecreção de ACTH Pituitária) OR (Hipersecreção Pituitária de ACTH) OR (Doença de Cushing) OR (Síndrome da Secreção Inadequada de ACTH) OR (Secreção Inadequada de Hormônio Adrenocorticotrópico) OR MH:C10.228.140.617.738.250.725$ OR MH:C19.700.355.800$

#2 MH: “Síndrome de Cushing” OR MH:C19.053.800.367$

#3 MH: “Gravidez” OR (Gestação) OR MH: G08.686.784.769

#1 AND #2 AND #3 = 15
